# Identification of a Novel Transcript and Regulatory Mechanism for Microsomal Triglyceride Transfer Protein

**DOI:** 10.1371/journal.pone.0147252

**Published:** 2016-01-15

**Authors:** Takashi Suzuki, Judy J. Brown, Larry L. Swift

**Affiliations:** 1 Department of Pathology, Microbiology and Immunology, Vanderbilt University School of Medicine, Nashville, Tennessee, United States of America; 2 Research Service, Veterans Affairs, Tennessee Valley Health Care System, Nashville, Tennessee, United States of America; International Centre for Genetic Engineering and Biotechnology, ITALY

## Abstract

Microsomal triglyceride transfer protein (MTP) is essential for the assembly of triglyceride-rich apolipoprotein B-containing lipoproteins. Previous studies in our laboratory identified a novel splice variant of MTP in mice that we named MTP-B. MTP-B has a unique first exon (1B) located 2.7 kB upstream of the first exon (1A) for canonical MTP (MTP-A). The two mature isoforms, though nearly identical in sequence and function, have different tissue expression patterns. In this study we report the identification of a second MTP splice variant (MTP-C), which contains both exons 1B and 1A. MTP-C is expressed in all the tissues we tested. In cells transfected with MTP-C, protein expression was less than 15% of that found when the cells were transfected with MTP-A or MTP-B. *In silico* analysis of the 5’-UTR of MTP-C revealed seven ATGs upstream of the start site for MTP-A, which is the only viable start site in frame with the main coding sequence. One of those ATGs was located in the 5’-UTR for MTP-A. We generated reporter constructs in which the 5’-UTRs of MTP-A or MTP-C were inserted between an SV40 promoter and the coding sequence of the luciferase gene and transfected these constructs into HEK 293 cells. Luciferase activity was significantly reduced by the MTP-C 5’-UTR, but not by the MTP-A 5’-UTR. We conclude that alternative splicing plays a key role in regulating MTP expression by introducing unique 5’-UTRs, which contain elements that alter translation efficiency, enabling the cell to optimize MTP levels and activity.

## Introduction

Microsomal triglyceride transfer protein (MTP)^2^ is a heterodimeric protein complex consisting of a unique 97 kD protein with lipid transfer activity and the ubiquitous 58 kD protein disulfide isomerase (PDI). The complex is essential for the assembly of triglyceride-rich, apolipoprotein (apo)B-containing lipoproteins [1], presumably transferring phospholipid, triglyceride, and cholesteryl ester from the endoplasmic reticulum (ER) membrane to the forming particle within the lumen of the ER. The genetic disease abetalipoproteinemia, characterized by a near absence of circulating triglyceride-rich lipoproteins, results from mutations in the gene encoding MTP, culminating in a loss of triglyceride transfer activity [2].

Given its role in the assembly of apoB-containing lipoproteins, it is not surprising that MTP is highly expressed in both liver and intestine [3, 4]. MTP has also been reported to be present in other apoB-expressing tissues, such as myocardium [5–7], retina [8], kidney [3, 9], yolk sac [10, 11], and placenta [12], highlighting the essential role of MTP in the transport of lipid out of the cell via apoB-containing lipoprotein complexes.

MTP is also expressed in tissues that do not express apoB such as ovary and testis [3]. The function of the protein in these tissues is unknown; however, both tissues are active in lipid metabolism, and MTP may serve specialized needs in lipid trafficking and/or storage in these cells. Our laboratory has shown that MTP is expressed in adipocytes [13, 14], where it is believed to be important in the formation and maturation of lipid droplets. In addition, MTP is expressed in professional antigen presenting cells (APCs) where it is required for the lipidation of the CD1 family of lipid antigen presenting proteins with both endogenous and exogenous lipids [15–17]. Furthermore, Dougan *et al*. have shown that MTP in thymocytes is critical to NKT cell development [17]. More recently MTP has been shown to be involved in the generation of antigenic lipid-CD1d complexes in adipocytes [18].

Previous studies have shown that mice express two isoforms of MTP [17, 19], which we designated MTP-A and MTP-B [19]. MTP-A is the canonical form. MTP-B is a splice variant with a unique first exon located ~2.7 kB upstream of the first exon of MTP-A. Both proteins have signal sequences that are cleaved on translocation into the ER. The mature proteins differ only in 2–3 amino acids in the N-terminus. Both proteins are equally effective in the assembly and secretion of apoB-containing lipoproteins [19]. MTP-A is the predominant isoform in liver and intestine based on mRNA levels, representing greater than 95% of total MTP mRNA in both tissues [19]. MTP-B appears to be the major isoform in adipocytes and 3T3-L1 cells [19], as well as in professional APCs [17].

In this paper, we report the identification of a second MTP splice variant in mice that contains exons 1B and 1A, a variant we have designated MTP-C. MTP-C is found in a number of tissues including brain, heart, lung, liver, kidney, testis, ovary, and fat. Whereas there are several potential translation initiation sites for this transcript, the only viable start site, in frame with the main open reading frame, is the start site for MTP-A. This results in an extended 5’-UTR, which we show suppresses translation efficiency and markedly decreases protein expression. We propose that upstream open reading frames (uORFs) in this 5’-UTR are important in the regulation of MTP, allowing the cell to fine tune MTP expression to meet the needs of the cell.

## Materials and Methods

### Antibodies and reagents

Female New Zealand White rabbits (Myrtle’s Rabbitry, Thompson Station, TN) were used for antibody production. All procedures performed on rabbits were reviewed and approved by the Institutional Animal Care and Use Committee (IACUC) and conducted in accordance with the Animal Welfare Act and the *Guide for the Care and Use of Laboratory Animals*. Rabbits were maintained in an AAALAC-accredited facility, singly housed in stainless steel cages in a controlled environment at 70 ± 2°F, 30–70% relative humidity, and a 12 h light/dark cycle. Food (Purina Maintenance Diet) and water were provided *ad libitum*. Fresh produce (depending on seasonal availability) and other environment enrichment aids, such as alfalfa cubes, pineapple spools, and chew toys, were provided on a periodic and rotating basis. The protocol for generating the antibody has been described previously [20]. In brief, the procedure was as follows. A 20-amino acid peptide (KYERLSTGRGYVSRRRKESC), representing residues 843 through 861 in the MTP protein plus an additional cysteine residue at the C-terminus was coupled to the carrier protein keyhole limpet hemocyanin (KLH) via the cysteine residue. An emulsion of the coupled peptide with the adjuvant TiterMax Gold® (Sigma Aldrich) was injected intradermally at multiple sites on the shaved and aseptically prepped backs of two rabbits. TiterMax Gold® was chosen over Freund’s adjuvant to reduce pain and distress to the animal. The rabbits were boosted with peptide coupled to BSA intradermally and intramuscularly after 5 weeks. Blood was collected 8 days after the boost. Humane endpoints were developed in consultation with university veterinarians and approved by IACUC. No animal met humane end point criteria.

The IgG fraction was isolated from serum using a protein-A column, and antibodies specific for the peptide were purified by passing the IgG fraction over a SulfoLink (Pierce, Rockford, IL) column-containing peptide, coupled according to the supplier's protocol. The concentration of the antibody was 0.66 μg/μl.

### Cell culture

CHO cells were purchased from American Type Culture Collection (Rockville, MD) and cultured in Kaighn’s modification of Ham’s F-12 (F12K) media containing 10% fetal bovine serum (FBS). HEK 293 cells were cultured in Dulbecco’s Modified Eagle’s Medium (Corning Life Sciences, Tewksbury, MA) supplemented with 10% FBS.

### Isolation of RNA and cDNA synthesis

Total RNA of tissues and cells was isolated using Trizol reagent (Life Technologies, Grand Island, NY) according to the manufacture’s instruction. cDNAs were synthesized using Super Script III Reverse Transcriptase (Life Technologies) according to the manufacturer’s instructions. In brief, 1 μg of total RNA was combined with random hexamers (Life Technologies) and oligo d(T)_16_ (Life Technologies) (both 1.25 μM final concentration) and dNTP mix (Sigma-Aldrich, 0.5 mM final concentration). The mixture was heated at 65°C for 5 min and then quickly chilled on ice for 5 min. 5X first strand reaction buffer, DTT (10 mM final concentration) and Super Script III enzyme (100 U) were then added, and the mixture was incubated at 25°C for 5 min, 50°C for 60 min, and 70°C for 15 min. To remove vector DNA from total RNA extracts, the extracts were treated with DNase I (New England Biolabs, Ipswich, MA) as follows. Total RNA (1 μg) was incubated with 2 unit DNase I (50 μl total volume) for 15 min at 37°C. DNase I treated RNA (5 μl) was used for the cDNA synthesis step.

### Primer Sequences for Cloning and Semi-Quantitative RT-PCR

Table 1 shows the source of cDNA and the primer sequences with the restriction enzyme sites used for PCR cloning of MTP genes. Easy-A High-Fidelity PCR Cloning enzyme (Agilent Technologies, Santa Clara, CA) was used to amplify full length MTP cDNAs. For PCR cloning, PCR cycle conditions were: 95°C for 30 s, 68°C for 10 min with 32 cycles. PCR products were ligated into pGEM-T easy vector (Promega, Madison, WI). After MTP gene sequences were confirmed, vectors were digested with restriction enzymes and full length MTP cDNAs were re-ligated into pcDNA3.1 (+) vector (Life Technologies).

**Table 1:** Source of cDNA and primer sequences for cloning mouse *Mttp* genes.

*Mttp* Gene	Primers	cDNA Source
MTP-A	Forward: tttaagcttaccATGATCCTCTTGGCAGTG (HindIII)	3T3-L1
	Reverse: ttgtctagaTCAAAACCATCCACCGGAGTTATC (XbaI)	
MTP-B, -C	Forward: tttaagcttaccATGACAGTCGTGATGGGGAAATG (HindIII)	MTP-B, 3T3-L1
	Reverse: ttgtctagaTCAAAACCATCCACCGGAGTTATC (XbaI)	MTP-C, brain

The primer sequences used for cloning uORFs of MTP are shown in Table 2. The forward primers contain a Hind III site, and the reverse primers contain an Nco I site used for subcloning into the pGL3-Control vector.

**Table 2:** Sequences of primer sets used for cloning uORFs of MTP.

*Mttp* uORF	Forward (Hind III)	Reverse (Nco I)
MTP-A	gtgaagcttATAAACACTGTTGTCGCCGG	taaccatggGCTGGCTCCCTCTGCCACAT
MTP-A + ATGA	gtgaagcttATAAACACTGTTGTCGCCGG	taaccatggtcatGCTGGCTCCCTCTGCC
MTP-A + ATAA	gtgaagcttATAAACACTGTTGTCGCCGG	taaccatggttatGCTGGCTCCCTCTGCC
MTP-C	gtgaagcttTGTTAACCGATTAACCTGGG	taaccatggGCTGGCTCCCTCTGCCACAT
MTP-C + ATGA	gtgaagcttTGTTAACCGATTAACCTGGG	taaccatggtcatGCTGGCTCCCTCTGCC
MTP-C + ATAA	gtgaagcttTGTTAACCGATTAACCTGGG	taaccatggttatGCTGGCTCCCTCTGCC

Table 3 shows the sequences of primer sets used for semi-quantitative RT-PCR. PCR cycle condition were: 95°C for 30 s, 60°C for 30 s, 72°C for 30 s with 35 cycles. Semi-quantitative RT-PCR was performed with GoTaq DNA polymerase.

**Table 3:** Sequences of primer sets used for semi-quantitative RT-PCR of mouse *Mttp* mRNA.

*Mttp* mRNA	Forward	Reverse
MTP-A	AGTGCTTTTTCTCTGCTTCTTCTC	ATTTTGTAGCCCACGCTGTC
MTP-B	TGCCGTGCTGTTACTCTTTC	ATTTTGTAGCCCACGCTGTC
MTP-C	GACGCCAGCTTTTGTTATTTT	CCTCTGCCACATCCAGTCC

### Transfections

Transfections were performed using FuGENE 6 transfection reagent (Promega, Madison, WI), according to the manufacturer’s instructions. In brief, in one well of a 6-well plate, 100 μl Opti-MEM (ThermoFisher Scientific, Grand Island, NY) and 3 μl FuGENE 6 were mixed then incubated for 5 min at room temperature. Vector DNA (1 μg) was added and incubated at room temperature for an additional 30 min. The FuGENE 6/DNA mixture was then added to cells in a dropwise manner.

### Isolation of Cell Protein

CHO and HEK 293 cells were solubilized in 20 mM HEPES (pH 7.4), 1.0 mM EGTA, 1% Triton X-100, and 10% glycerol on ice for 20 min. The extracts were then centrifuged at 4°C for 5 min at 14,000 x g in an Eppendorf microfuge. The supernatant was recovered, and protein concentration was determined using the bicinchoninic acid (BCA) method (ThermoFisher Scientific). Aliquots were taken for SDS-PAGE and immunoblotting as described below.

### SDS-Polyacrylamide Gel Electrophoresis and Immunoblotting

Samples were solubilized in NuPAGE LDS sample buffer and separated by SDS-PAGE using NuPAGE bis-tris gels (4–12% gradients) (Life Technologies) with morpholinepropanesulfonic acid SDS running buffer [21]. The proteins were transferred to nitrocellulose membranes. The membranes were blocked in TBS with 5% non-fat milk, incubated overnight at 4°C with primary antibody, washed extensively, and incubated for 1 h at room temperature with the appropriate secondary antibody conjugated with horseradish peroxidase. Bands were visualized using Western Lightning® Plus-ECL enhanced chemiluminescence substrate (Perkin Elmer, Waltham, MA) and quantitated by densitometry (BioRad Model GS-700 Imaging Densitometer) equipped with Quantity One software.

### Triglyceride Transfer Activity

Triglyceride transfer activity was assessed using a fluorescent-based kit assay [22] (Chylos, Inc., Woodbury, NY). CHO cells were transfected with mouse MTP-A, -B, or -C as described above. Three days post transfection the cells were lysed, and aliquots of the lysate were used in the transfer assay and for protein determination (BCA). The transfer assays were run for different times (0.5 to 5 h), and the results were expressed as the percent triglyceride transferred per 10 μg of cell protein. Lysate from HepG2 cells was used as a positive control. CHO cells transfected with an empty pcDNA vector served as a negative control.

### Translation Luciferase Assays

The following 5’-untranslated regions (UTRs) were subcloned downstream of an SV40 promoter and upstream of the firefly luciferase gene in a pGL3-Control vector (Promega): 1) 5’-UTR for MTP-A; 2) 5’-UTR for MTP-A plus first four bases (ATGA) of coding sequence of MTP; 3) 5’-UTR for MTP-C; 4) 5’-UTR for MTP-C plus first four bases (ATGA of coding sequence of MTP. HEK 293 cells were transfected with pGL3-Control or one of the 5’-UTR pGL3 reporter constructs (above), along with pRL-TK renilla luciferase construct (Promega) using FuGENE 6. The cells were harvested three days after transfection and assayed for luciferase activity using a Dual-Glo Luciferase Assay System (Promega).

### Ethics Statement

This study was carried out in strict accordance with the recommendations in the Guide for the Care and Use of Laboratory Animals of the National Institutes of Health. The protocol was approved by the Institutional Animal Care and Use Committee (IACUC) of Vanderbilt University (Protocol Number: V/14/025).

## Results

### Discovery of MTP-C

**Fig 1:**
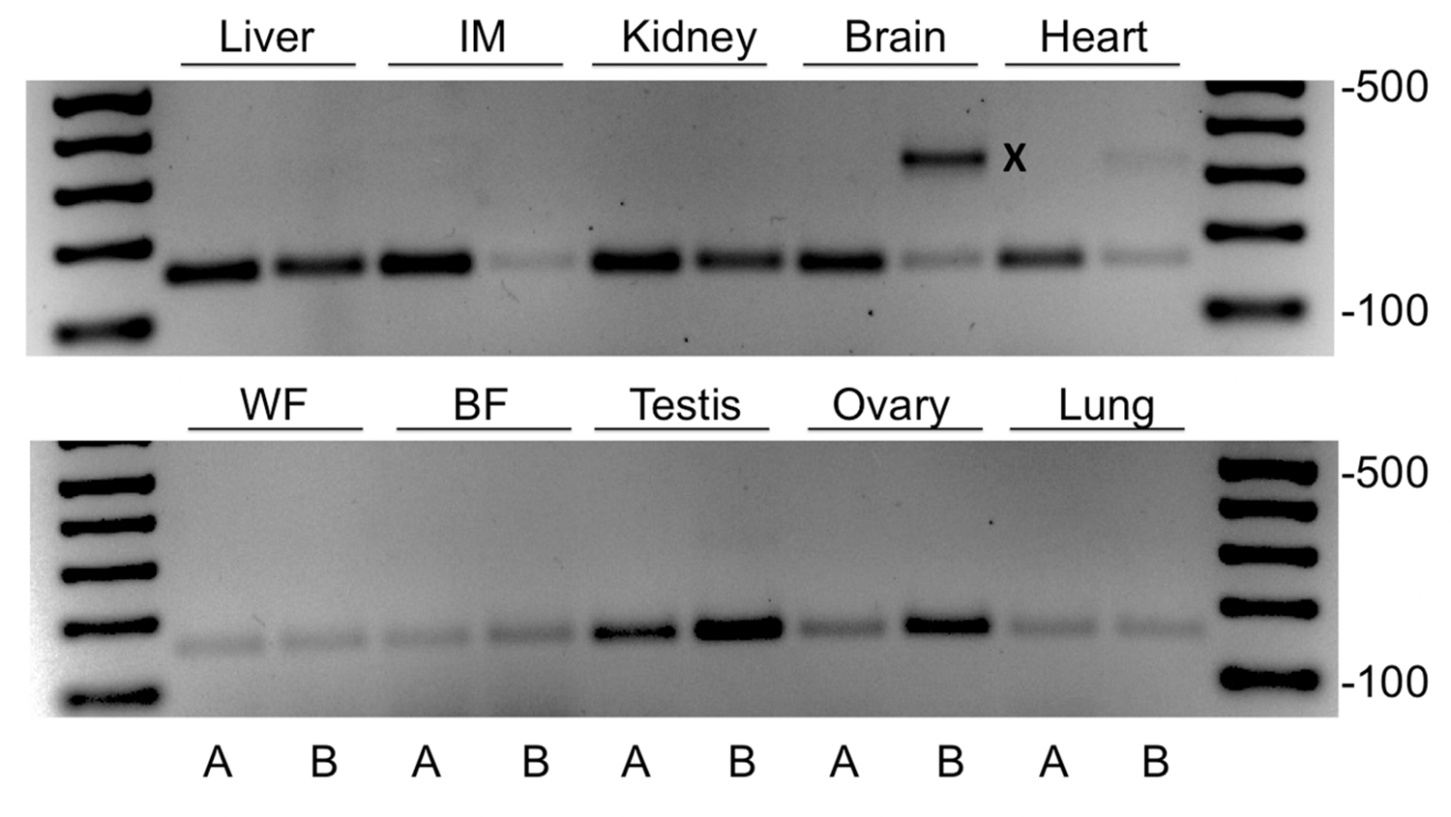
Identification of MTP-C in mouse tissue. RNA was extracted from mouse tissues, and RT-PCR was run using forward primers in exons 1A and 1B and a reverse primer in exon 2. Specific fragments were generated for MTP-A and MTP-B in all tissues examined. Note the extra product in brain tissue (X) when using primers for MTP-B. IM, intestinal mucosa; WF, white fat; BF, brown fat.

**Fig 2:**
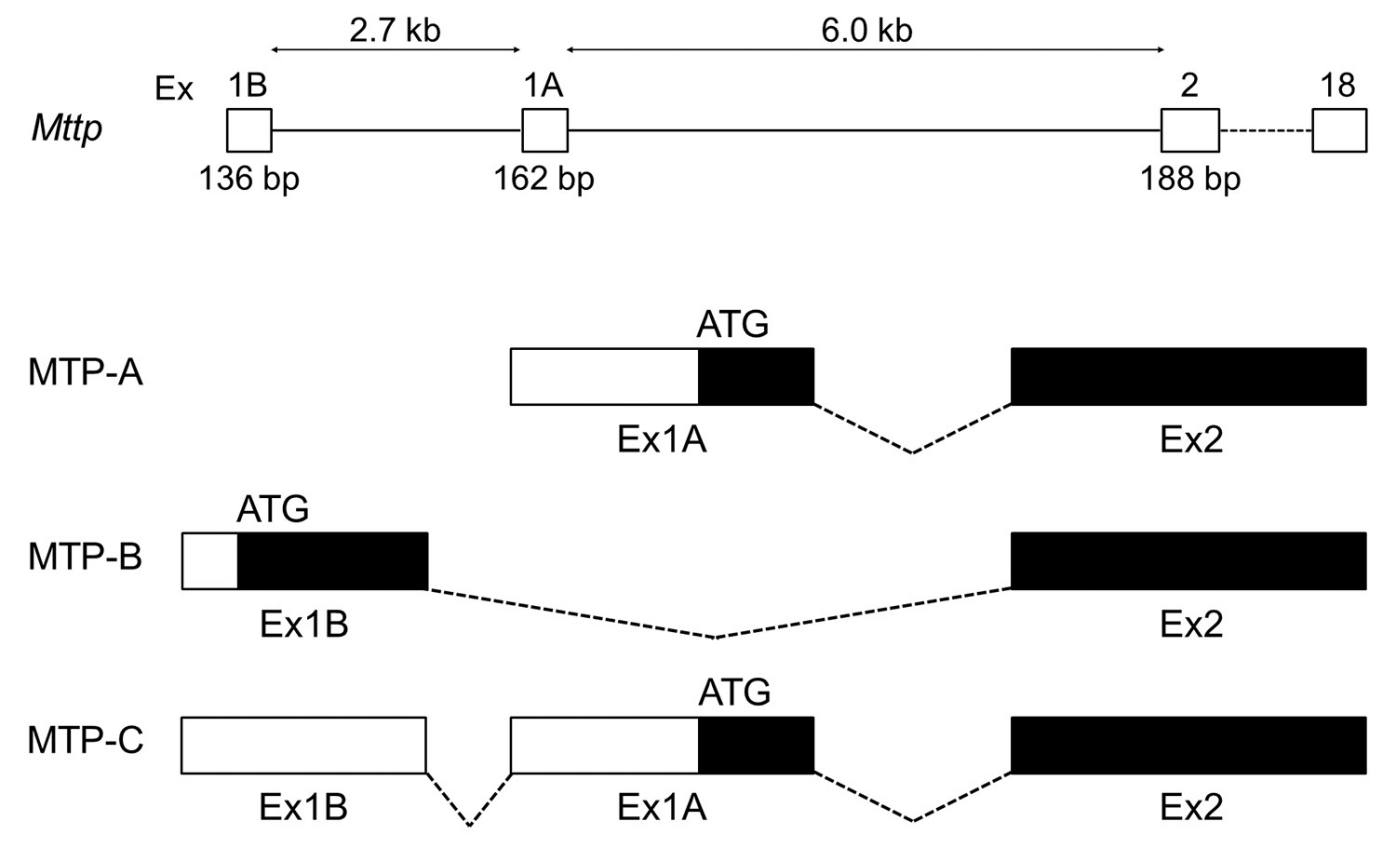
Comparison of mouse MTP splice variants. The mouse *Mttp* gene consists of 19 exons spanning 40 kB. Exon 1B is located 8.7 kB upstream of exon 2 and 2.7 kB upstream of exon 1A, which contains the initiator codon for MTP-A. MTP-B arises through a novel mechanism in which exon 1B is spliced directly to exon 2. The MTP-C transcript arises when exon 1A is not spliced out of the MTP-B transcript; thus, it contains both exons 1B and 1A. Black areas in exons represent open reading frames; white areas represent 5’-untranslated regions (5’-UTR).

While probing the relative expression levels of MTP-A and -B mRNA in a number of mouse tissues using forward primers in exons 1A and 1B, respectively, and a reverse primer in exon 2, we generated fragments specific for MTP-A and MTP-B in all tissues examined (Fig 1). In addition, we noted a prominent fragment (X) in brain tissue that was 150–200 nucleotides larger than the fragment generated for MTP-B. We sequenced the larger fragment and found that it corresponded to a transcript in which exon 1A had not been spliced out of the primary transcript, a process that would normally occur in the production of MTP-B mRNA. Consequently, the transcript included both exons 1B and 1A (Fig 2). We named this new variant, MTP-C. The identification of the start site for this transcript is discussed below.

In the sequencing of the MTP-C fragment, we discovered that the published record for mouse Mttp mRNA (transcript variant 2 or MTP-A, NM_008642) was incomplete with regard to exon 1 (1A), missing 24 bases at the 5’ end. We examined the genomic sequence and confirmed that exon 1 consists of 162 bases (not 138), with the extra 24 bases being identical to what we found in sequencing the MTP-C fragment. Thus, the size of exon 1 for mouse MTP is identical to exon 1 for human MTP.

### Tissue Expression of MTP-C mRNA

**Fig 3:**
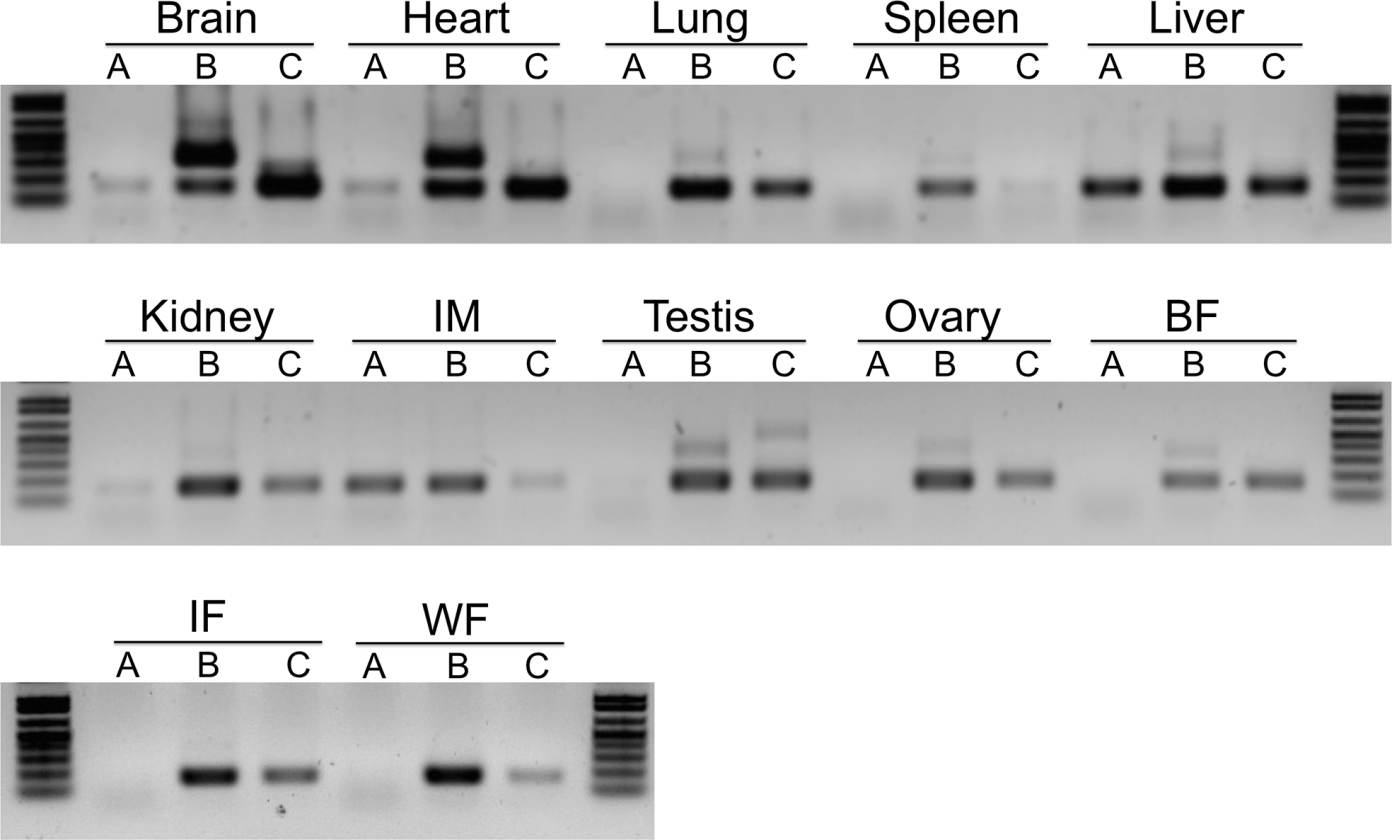
Expression of MTP splice variants in mouse tissues. The expression of MTP-A, -B, and -C was probed in a number of mouse tissues. MTP-C was found in all tissues examined. It was robustly expressed in brain, heart, liver and testis. IM, intestinal mucosa; BF, brown fat; IF, inguinal fat; WF, white fat.

To probe mouse tissues for MTP-C, we developed MTP-C specific primers with the forward primer located in exon 1B and the reverse primer in exon 1A. For comparison we monitored the expression of MTP-A and MTP-B (Fig 3). MTP-C was present in all tissues tested. It was especially prominent in brain, heart, liver, and testis.

### Expression of MTP-C in CHO and HEK 293 cells

**Fig 4:**
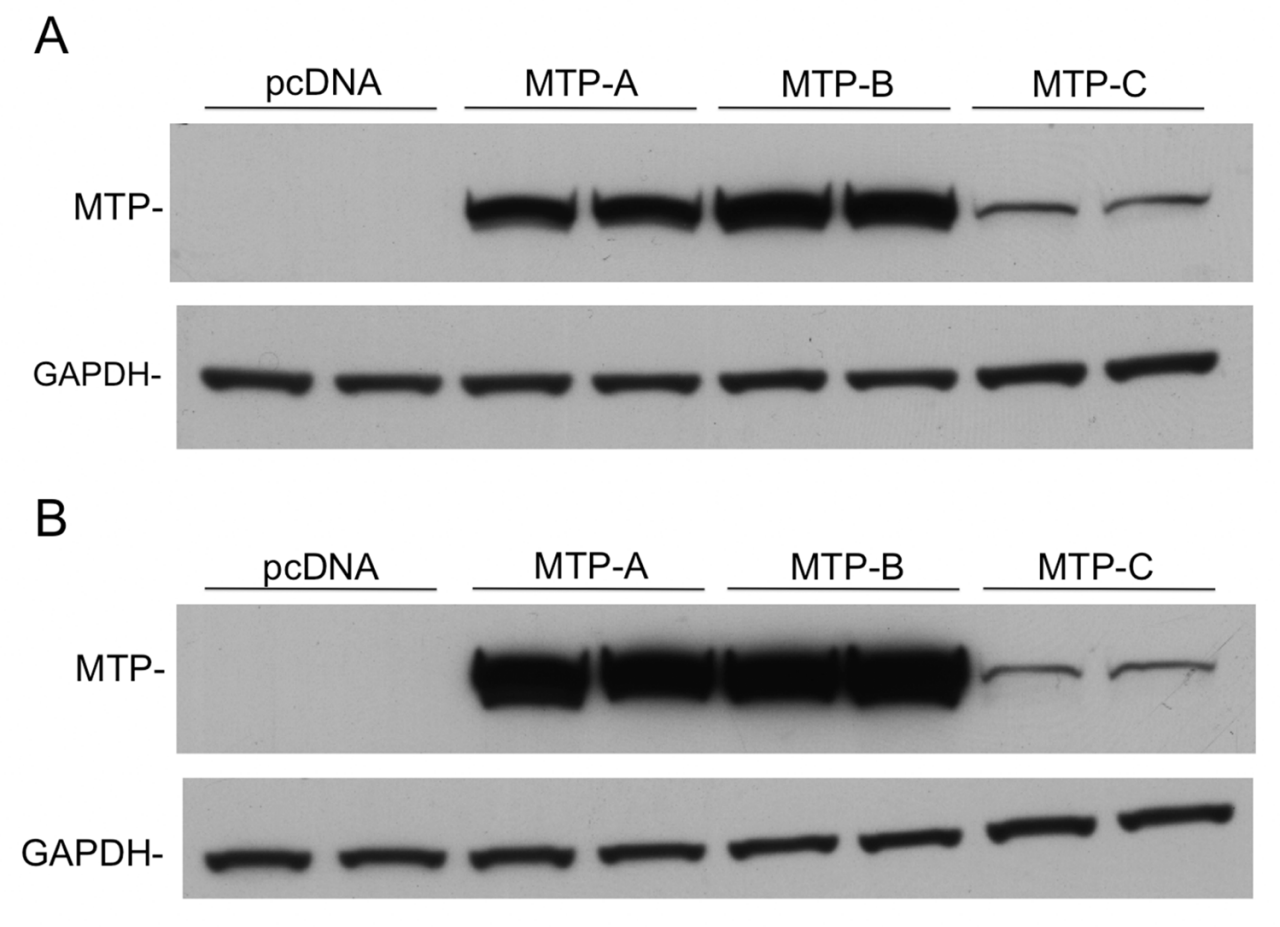
Translation efficiency of MTP splice variants. Full-length MTP-A, MTP-B, and MTP-C were sub-cloned into pcDNA3.1 vectors and transfected into CHO cells (A) and HEK 293 cells (B). Seventy-two hours post transfection, equal amount of cell lysate protein were separated by SDS-PAGE, and MTP protein levels were assessed by immunoblotting. The films were scanned using a BioRad GS700 Imaging Densitometer equipped with Quantity One software to assess levels of MTP protein relative to GAPDH.

MTP-A, MTP-B, and MTP-C were cloned into pcDNA3.1, and equivalent amounts of DNA were transfected into CHO cells and HEK 293 cells. Three days after transfection, cellular MTP levels were assessed by immunoblotting (Fig 4). In CHO cells, MTP-A and MTP-B were robustly expressed with MTP-B expression slightly greater than MTP-A (Fig 4A). In contrast, MTP expression in cells transfected with the MTP-C transcript was approximately 15% of MTP-A and 11% of MTP-B levels. MTP-A and MTP-B expression in HEK 293 cells appeared to be even more robust than in CHO cells, with both proteins expressed at nearly equal levels (Fig 4B). MTP expression in HEK 293 cells transfected with MTP-C was approximately 5% of the levels seen in cells transfected with either MTP-A or -B. To eliminate the possibility that the decrease in protein expression observed in cells transfected with MTP-C was related to transfection efficiency of the transcript and/or mRNA production, we measured MTP mRNA levels in CHO cells transfected with each of the transcripts after DNase treatment (S1 Fig). As can be seen there was no difference in MTP mRNA levels between the cells transfected with the different transcripts.

### Triglyceride Transfer Activity

**Fig 5:**
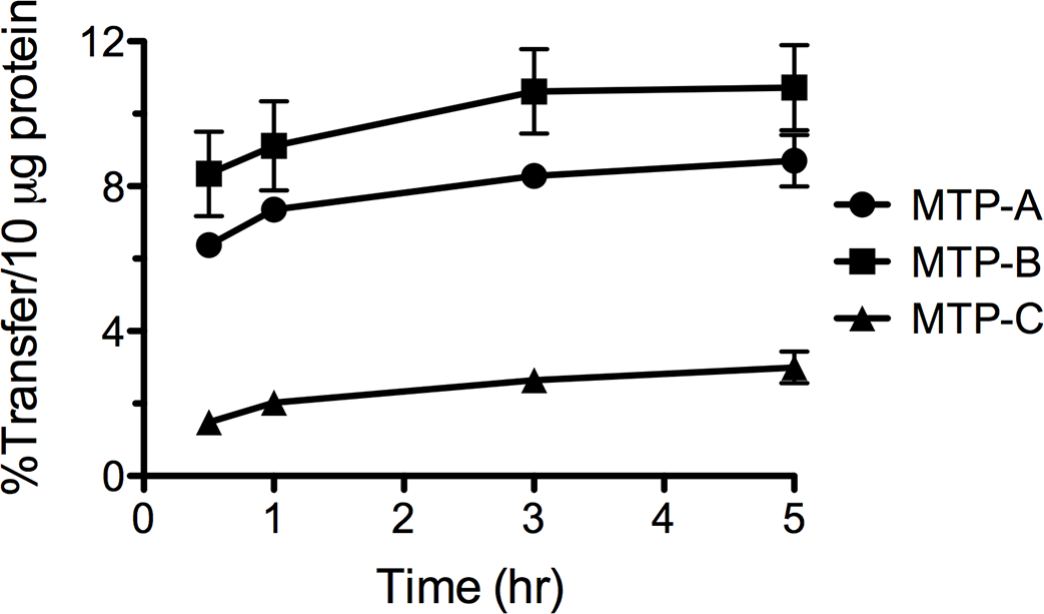
Triglyceride transfer activity. CHO cells were transfected with mouse MTP-A, -B, and -C. Three days post transfection the cells were lysed and aliquots of the lysate taken for the fluorescence based triglyceride transfer assay (Chylos, Inc.). The assays were run for varying periods, and the results are expressed as the percent of total triglyceride transferred/10 μg cell protein. Data are expressed as mean ± s.d. (n = 3/group).

Triglyceride transfer activity in CHO cells transfected with MTP-A, MTP-B and MTP-C was evaluated using a fluorescence based assay (Fig 5). Triglyceride transfer activity was detected in cell lysates from each of the constructs at all time points. The transfer activity in cells transfected with MTP-B was slightly higher (~30%) at each time point than that found in cells transfected with MTP-A; however, the difference paralleled almost perfectly the difference in MTP protein expression observed in CHO cells transfected with the two variants (Fig 4). Importantly, the experiments demonstrated that transfection with MTP-C leads to expression of a functional protein. The transfer activity in cells transfected with MTP-C was significantly reduced at all time points compared with MTP-A and MTP-B and parallels the relative protein levels observed in the cells (Fig 4).

### Analysis of 5’-untranslated region

**Fig 6:**
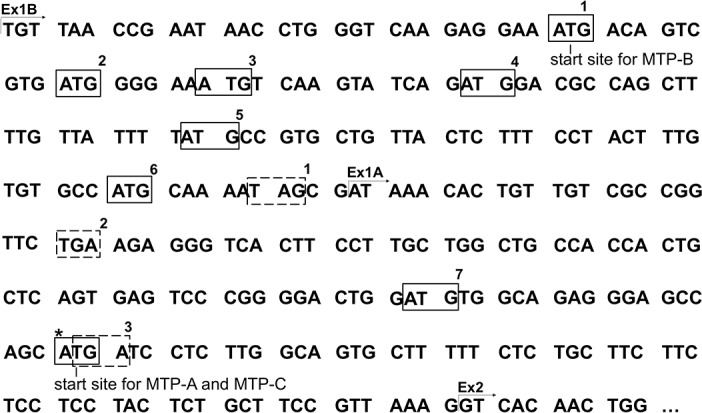
Nucleotide sequence for exons 1B and 1A. Start (boxes with solid lines) and stop (boxes with dashed lines) codons in the 5’-UTR of MTP-C are highlighted. Start sites ATG-1, -2, and -6 are in frame with a TGA-2; ATG-3 is in frame with TAG-1; ATG-4, -5, and -7 are in frame with TGA-3. ATG* denotes translation initiation site for MTP-A. Note that this start site codon overlaps a potential stop codon.

To explore why transfection with MTP-C leads to markedly reduced levels of MTP protein, we focused on the 5’-UTR of the transcript. Examination of this region revealed seven ATGs before the actual start site for MTP-A (ATG*) (Fig 6). Three of these start sites in exon 1B (ATG-1, -2, -6) are in frame with the main coding sequence; however, they are also in frame with a stop codon in exon 1A (TGA-2) that would halt translation. The remaining four ATGs (ATG-3, -4, -5, -7) are out of frame with the main coding sequence. ATG-3 in exon 1B is in frame with a stop codon in exon 1B (TAG-1), prior to the main coding sequence. Three start sites (ATG-4, -5, -7) are in frame with a stop codon (TGA-3) that overlaps with the translation initiation site for MTP-A (ATG*). Thus, within the 5’-UTR, there are seven potential open reading frames (ORFs) ranging in size from 21 to 174 nucleotides. The only viable start site for translation of MTP-C is the same start site for MTP-A (ATG*) (See also Fig 2).

### Translation Efficiency as Assessed with Luciferase Assays

**Fig 7:**
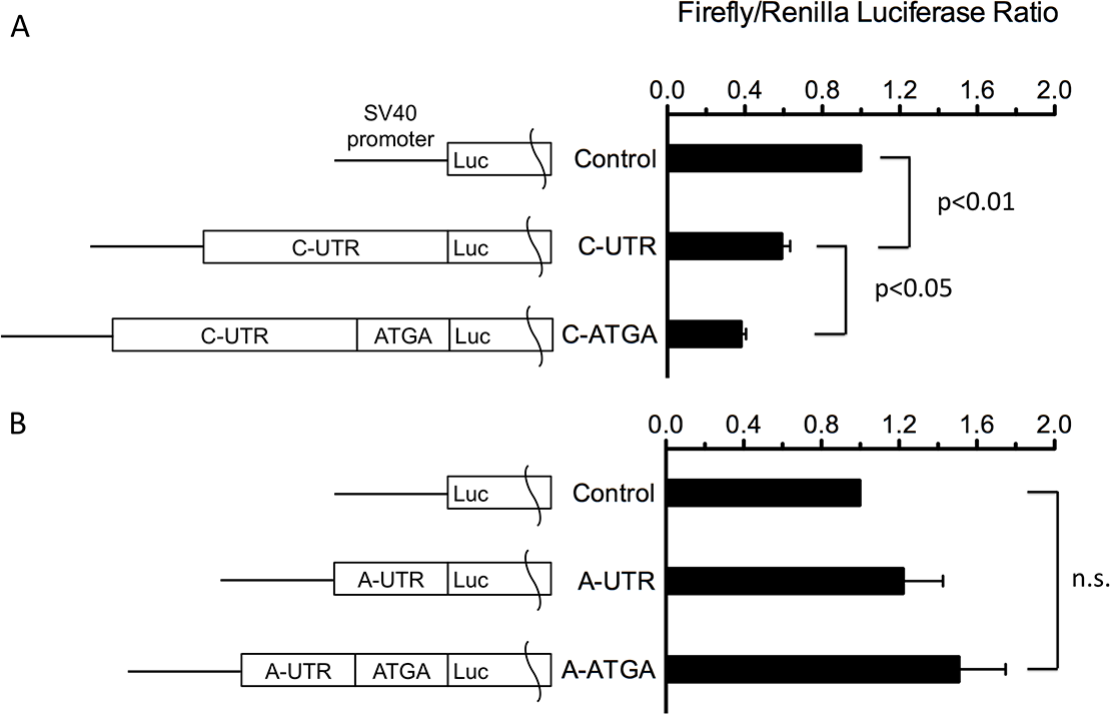
Effects of 5’-UTR of MTP-A and MTP-C on translation efficiency. CHO cells were transfected with Renilla luciferase and 5’-UTR constructs of either MTP-C (top) or MTP-A (bottom). The cells were lysed and assayed for luciferase activity. Data are expressed as mean ± s.d. (n = 3/group). a, significantly different from Control, p < .001; b, significantly different from C-UTR, p < .05.

To determine if the 5’-UTR of MTP-C regulates translation efficiency of MTP, we subcloned the 217 nucleotide 5’-UTR of MTP-C into the pGL3 firefly luciferase vector downstream of the SV40 promoter. In addition, we subcloned the 101 nucleotide 5’-UTR of MTP-A into the firefly luciferase vector; the empty vector served as a control. The vectors were transfected into HEK 293 cells, and luciferase activity was measured in cell lysates three days after transfection. Luciferase activity was significantly reduced (40.7 ± 7.1%, p< 0.001) in cells transfected with the 5’-UTR of MTP-C compared with pGL3-Control (Fig 7). Interestingly, when the first four nucleotides of the main coding sequence (CDS) were included with the 5’-UTR, providing a stop codon (TGA-3, Fig 6) for three of the upstream translation initiation sites (ATG-4, -5, -7, Fig 6), luciferase activity was reduced an additional 35.3 ± 2.3% (p < .05). In cells transfected with the 5’-UTR of MTP-A there were no significant changes in luciferase activity, although there was a trend toward increased activity with each of the constructs.

### Effect of 5’-UTR on cellular expression of MTP-A

**Fig 8:**
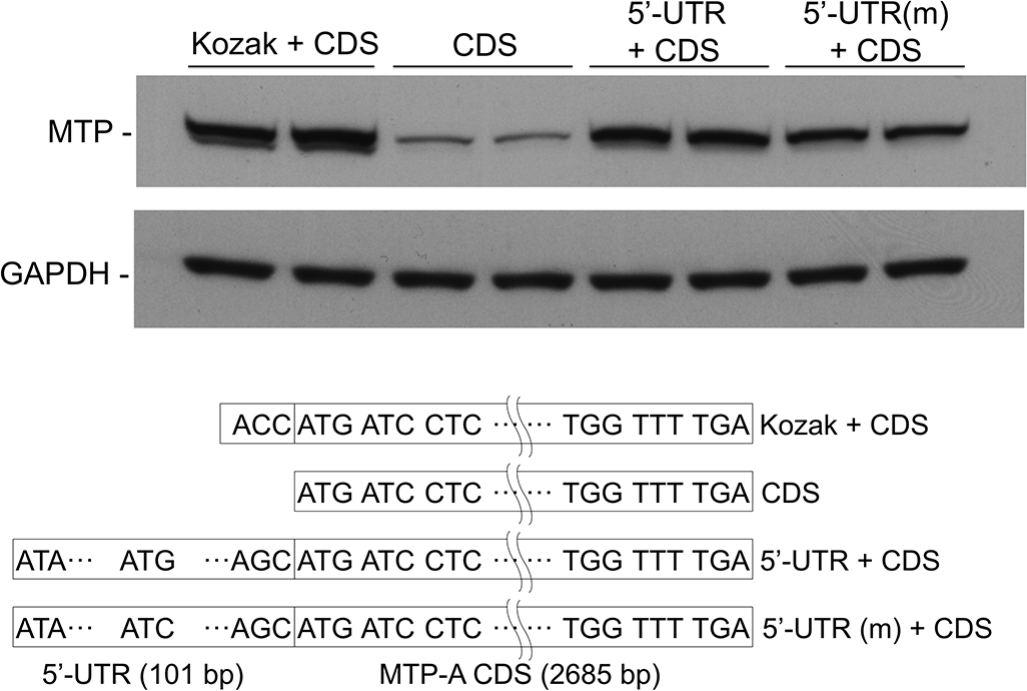
Effect of 5’-UTR on expression of MTP-A. CHO cells were transfected with MTP-A open reading frame (CDS), MTP-A ORF with artificial Kozak sequence (Kozak + CDS), MTP-A ORF with full 5’-UTR (5’-UTR + CDS), and MTP-A ORF with full 5’-UTR in which upstream ATG was mutated to ATA (5’-UTR(m) + CDS). Cells were lysed 72 h post transfection, and equal amounts of cell lysate protein were separated by SDS-PAGE. MTP protein levels were assessed by immunoblotting and quantitated using a BioRad GS700 Imaging Densitometer equipped with Quantity One software.

To test the effect(s) of the 5’-UTR on cellular expression of MTP-A, we transfected CHO cells with MTP-A constructs that contained the CDS with and without the 5’-UTR (Fig 8). Addition of the 5’-UTR to the CDS increased protein expression to a level similar to that seen when a Kozak consensus sequence (ACC) was added immediately before the ATG in the CDS (Kozak + CDS), (Fig 8). This suggested that enhancement of protein expression in the presence of the 5’-UTR might result from the presence of a Kozak consensus sequence at the translation initiation site. Interestingly, when the uATG was mutated to ATA (5’-UTR(m)), abolishing a potential uORF, MTP protein expression decreased 42.7 ± 14.7% (n = 3). This suggests that the enhancement in translation induced by the addition of the 5’-UTR is not totally dependent on the presence of the Kozak consensus sequence, but is at least partly related to additional elements within the 5’-UTR, e.g., uORF.

## Discussion

Previous studies in our laboratory led to the identification of a splice variant of mouse MTP, which we designated MTP-B [19]. MTP-B has a unique first exon (1B) located ~2.7 kB upstream of exon 1(A) for canonical MTP, which we designated MTP-A. Both proteins are synthesized with signal sequences that are quite different; however, the two mature proteins differ only in the N-terminal 2–3 amino acids, and both function equally well in the assembly of apoB-containing triglyceride-rich lipoproteins [19]. However, the tissue distribution of the two variants as assessed by mRNA level varies widely from tissue to tissue. Liver and small intestine express primarily the MTP-A variant, whereas the MTP-B variant is more prominent in adipose tissue [19].

In this paper we report the discovery of a second splice variant of MTP in which exon 1A is not spliced out of the transcript that would normally produce MTP-B, resulting in a transcript containing both exons 1B and 1A (Fig 2). In keeping with our nomenclature, we have named this splice variant MTP-C. MTP-C, which is expressed in several tissues (Fig 3), encodes canonical MTP (or MTP-A); however, MTP protein production in cells transfected with this transcript is markedly suppressed compared to cells transfected with either MTP-A or -B transcripts (Fig 4). To understand the decreased protein expression with the MTP-C splice variant we focused on the 5’-UTR, as this was the major difference between MTP-C and MTP-A/MTP-B splice variants. Luciferase experiments demonstrated clearly that the 5’-UTR of MTP-C was associated with decreased translation compared with MTP-A (Fig 7). Examination of the 5’-UTR sequence of MTP-C revealed seven potential uORFs ranging in size from 21–174 nucleotides (Fig 6). uORFs are defined by a start codon in the 5’-UTR that is in frame with a stop codon located either upstream or downstream of the main coding sequence initiation site. Numerous studies have shown that uORFs are generally, but not always [23], associated with decreased protein expression with reductions estimated to range from 30 to 80% [24]. The reduction in protein expression is thought to be related to the translation efficiency of the downstream ORF. For an uORF to function as a translational regulatory element, its translation initiation site must be recognized by the ribosome, and this is dependent on the strength of the consensus Kozak sequence [25]. In the case of weaker sequences, leaky scanning can occur in which the ribosome can either read the ATG or scan by it to initiate translation at a start site further downstream. If it is recognized it may (1) translate the uORF and dissociate, (2) translate the uORF and stall, creating a blockade to additional ribosome scanning or triggering mRNA decay, or 3) translate and then re-initiate to translate the downstream ORF. In any case, translation efficiency is compromised, and protein expression is decreased.

Our finding that the 5’-UTR of MTP-C contains elements that play a role in regulating protein expression led us to examine the possibility that the 5’-UTR may play a role in regulating the expression of canonical MTP (or MTP-A). The 5’-UTR for MTP-A consists of 101 nucleotides with a single ATG (ATG-7, Fig 6) located 17 nucleotides upstream of the MTP-A translation initiation site. Importantly, the MTP-A 5’-UTR did not suppress translation as was observed with the 5’-UTR for MTP-C. In fact, we observed a trend toward increased translation efficiency with the MTP-A 5’-UTR, although the increase was not statistically significant (Fig 7). However, additional experiments provided evidence that the 5’-UTR in general and its one uATG in particular enhanced MTP-A protein expression (Fig 8). Transfection of cells with an MTP-A construct containing the complete 5’-UTR led to a dramatic increase in MTP protein expression compared with cells transfected with the coding sequence for MTP-A. Undoubtedly, this increase in protein expression results, in part, from the presence of nucleotides that increase the strength of the Kozak sequence surrounding the authentic translation initiation site. In fact, the addition of an artificial Kozak consensus sequence to the construct containing only the MTP-A coding sequence leads to MTP protein levels similar to that observed with the 5’-UTR MTP-A construct (Fig 8). It is, however, important to note that mutation of the uATG to ATA leads to a 42% decrease in MTP levels (Fig 8). Our results demonstrate that the 5’-UTRs of MTP-C and MTP-A function in opposite directions in the regulation of MTP protein levels. In the case of MTP-C, elements in the 5’-UTR serve to suppress translation, whereas in MTP-A, elements in the 5’-UTR enhance translation. Together the results point to the importance of alternative splicing in the overall regulation of MTP levels.

Alternative splicing can impact cellular protein expression in a number of ways. It can: 1) alter the main ORF leading to changes in the sequence of the protein; 2) lead to changes in mRNA stability, which in turn leads to nonsense mediated decay (NMD) of the mRNA product; or 3) it can have important regulatory consequences affecting translatability by producing transcripts with similar ORFs but different 5’ or 3’ UTRs. A good example is the human angiotensin II type I receptor (AT_1_R). A number of studies have shown at least four distinct mRNA splice variants, representing combinations of four exons, are synthesized from the single AGTR1 gene [26–30]. The main ORF is contained in the last exon [26, 30]; the presence of exon 1 or exon 2 markedly inhibits translation [27, 28]. The inhibitory effect of exon 1 is believed to be due to secondary structure that is too stable to be efficiently unwound by 40S ribosomes [27, 28]. Inhibition by exon 2 is believed to be related to an upstream ORF [27, 28] as it contains a translation start site in optimal context for initiation of translation followed by a stop codon 21 nucleotides downstream.

Numerous studies have suggested that MTP levels are controlled via transcriptional, post transcriptional and post translational mechanisms as well as by nutritional and hormonal factors (reviewed by Hussain *et al*. [31]). In addition, Soh *et al*. have recently shown that MTP is regulated by microRNAs, specifically by members of the miRNA-30c family [32]. Micro RNA 30c (miR 30c) represses MTP expression by targeting a conserved site in the 3’-UTR of *mttp* mRNA. One might question that with all the other mechanisms for regulating MTP expression, what is significant about regulation via uORFs? Upstream ORFs and ATGs are common in certain classes of genes, including two-thirds of oncogenes and other genes involved in control of cellular growth and differentiation [33–35]. They are not uncommon in genes with critical cellular roles [36]. Recently Cadar *et al*. [37] provide evidence that titin translational efficiency is controlled in part by its 5’-UTR, mainly through a *cis*-regulatory uORF. Titin is the largest known protein, a critical determinant of myofibril elasticity and sarcomere structure in striated muscle. Cadar *et al*. [37] hypothesize that the uORF plays a role in fine tuning mRNA translation by serving as a passive brake to prevent overproduction and hence the wasting of cellular resources, given that the synthesis of this mega-protein is energetically costly.

MTP has long been known to be essential for the assembly of triglyceride-rich apoB-containing lipoproteins. In addition, we are discovering that MTP is expressed in a number of tissues. While we do not know the precise functions of MTP in these tissues, we presume it plays some specialized role in the transport of lipids within the cell. We do know that within certain tissues the level of MTP activity is critical for the maintenance of normal cell physiology and function. A case in point is the liver. Pharmacologic inhibition of MTP activity leads to decreased secretion of apoB-containing, triglyceride-rich lipoproteins by HepG2 [38] cells as well as in rabbits [39] and humans [40]. However, decreased MTP activity can also lead to varying degrees of hepatic steatosis. In contrast, hepatic overexpression of MTP leads to hypertriglyceridemia caused by increased secretion of VLDL [41]. Thus, the level of MTP activity within the hepatocyte is critical in maintenance of normal cellular physiology with negative consequences when the levels are either too low or too high. In addition, the levels of MTP required for normal cellular function may vary from cell to cell. For example, in the mouse deletion of *Mttp* is incompatible with embryonic development, presumably because MTP is essential for the transport of nutrients from the yolk sac to the developing embryo [10]. In contrast, embryonic development in *Mttp*^*+/-*^ mice is normal despite a 50% reduction in *Mttp* mRNA; however, hepatic lipid/lipoprotein metabolism is altered in these mice [10]. Thus, the mechanisms underlying the regulation of MTP expression must be such as to maintain levels within an optimal range, avoiding consequences of too little or too much MTP. We speculate that uORFs may provide a mechanism by which tissue levels of MTP can be fine-tuned to provide maximal function.

In conclusion, we have identified a new splice variant of mouse MTP. This new variant, which we have named MTP-C, contains both exons 1B and 1A, the first exons for MTP-B and MTP-A, respectively. Whereas there are several potential start sites in exons 1B and 1A, the only viable start site is for MTP-A. Importantly, we have shown that the extended 5’-UTR for MTP-C contains elements that suppress translation and MTP protein production. In contrast, the 5’-UTR for MTP-A enhances protein expression. The results not only demonstrate the importance of the 5’-UTR, but also the importance of alternative splicing in the regulation of MTP expression. We speculate that this mechanism provides a way for tissues to “fine tune” the expression of MTP, avoiding the negative consequence of either too much or too little MTP.

## Supporting Information

S1 Fig MTP mRNA levels in CHO cells transfected with different MTP constructs. CHO cells were transfected with MTP-A (mA), MTP-B (mB), or MTP-C (mC). Three days after transfection, total RNA was isolated. The RNA extract was treated with DNase I as described in Materials and Methods. MTP mRNA levels were assessed by RT-PCR using the following primers: Forward—TATGGAGATCCAGGGTGGTC; Reverse—CTGCTTTCCACACCAGCTTT. Primer sequences for β-actin were: Forward—AGCCATGTACGTAGCCATCC; Reverse—CTCTCAGCTGTGGTGGTGAA.
(TIF)
