# Supplementary figures and images for: Identification of a Novel Transcript and Regulatory Mechanism for Microsomal Triglyceride Transfer Protein
Source: PLoS One. 2016 Jan 15;11(1):e0147252. doi: 10.1371/journal.pone.0147252 (PMC4714884; doi:10.1371/journal.pone.0147252)

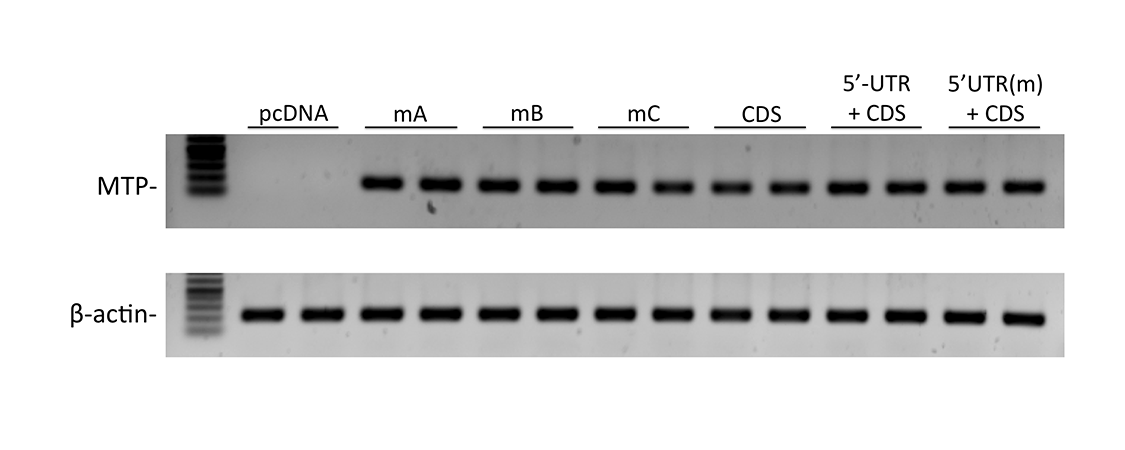

Supplement: S1 Fig — CHO cells were transfected with MTP-A (mA), MTP-B (mB), or MTP-C (mC). Three days after transfection, total RNA was isolated. The RNA extract was treated with DNase I as described in Materials and Methods. MTP mRNA levels were assessed by RT-PCR using the following primers: Forward—TATGGAGATCCAGGGTGGTC; Reverse—CTGCTTTCCACACCAGCTTT. Primer sequences for β-actin were: Forward—AGCCATGTACGTAGCCATCC; Reverse—CTCTCAGCTGTGGTGGTGAA. (TIF) [file pone.0147252.s001.tif]
